# DNA methylome and transcriptome sequencing in human ovarian granulosa cells links age-related changes in gene expression to gene body methylation and 3ʹ-end GC density

**DOI:** 10.18632/oncotarget.2875

**Published:** 2015-02-17

**Authors:** Bo Yu, Valya R. Russanova, Silvia Gravina, Stephen Hartley, James C. Mullikin, Alice Ignezweski, James Graham, James H. Segars, Alan H. DeCherney, Bruce H. Howard

**Affiliations:** ^1^ Department of Obstetrics and Gynecology & Women's Health, Albert Einstein College of Medicine, Bronx, New York 10461, USA; ^2^ Program in Genomics of Differentiation, Eunice Kennedy Shriver National Institute for Child Health and Human Development, National Institutes of Health, Bethesda, Maryland 20892, USA; ^3^ Department of Genetics, Albert Einstein College of Medicine, Bronx, New York 10461, USA; ^4^ Comparative Genomics Unit, Genome Technology Branch, National Human Genome Research Institute, National Institutes of Health, Bethesda, Maryland 20892, USA; ^5^ NIH Intramural Sequencing Center, National Human Genome Research Institute, National Institutes of Health, Bethesda, Maryland 20892, USA; ^6^ Shady Grove Fertility Reproductive Science Center, Rockville, Maryland 20850, USA; ^7^ Program in Reproductive and Adult Endocrinology, Eunice Kennedy Shriver National Institute for Child Health and Human Development, National Institutes of Health, Bethesda, Maryland 20892, USA

**Keywords:** DNA methylation, transcription end site, fertility, ovarian granulosa cell, transcriptome

## Abstract

Diminished ovarian function occurs early and is a primary cause for age-related decline in female fertility; however, its underlying mechanism remains unclear. This study investigated the roles that genome and epigenome structure play in age-related changes in gene expression and ovarian function, using human ovarian granulosa cells as an experimental system. DNA methylomes were compared between two groups of women with distinct age-related differences in ovarian functions, using both Methylated DNA Capture followed by Next Generation Sequencing (MethylCap-seq) and Reduced Representation Bisulfite Sequencing (RRBS); their transcriptomes were investigated using mRNA-seq. Significant, non-random changes in transcriptome and DNA methylome features are observed in human ovarian granulosa cells as women age and their ovarian functions deteriorate. The strongest correlations between methylation and the age-related changes in gene expression are not confined to the promoter region; rather, high densities of hypomethylated CpG-rich regions spanning the gene body are preferentially associated with gene down-regulation. This association is further enhanced where CpG regions are localized near the 3ʹ-end of the gene. Such features characterize several genes crucial in age-related decline in ovarian function, most notably the *AMH* (Anti-Müllerian Hormone) gene. The genome-wide correlation between the density of hypomethylated intragenic and 3ʹ-end regions and gene expression suggests previously unexplored mechanisms linking epigenome structure to age-related physiology and pathology.

## INTRODUCTION

Since the first assisted conception in 1978, assisted reproductive technologies (ART) have enabled infertile couples to give birth to 4 million children. Assisted reproductive technologies (ART) have now circumvented most etiologies of male and female infertility; however, it has low success in overcoming subfertility related to female age. Even with ART, only 12.2% of women between age 41 and 42, or 4.2% of women older than 42 achieve live births with their own oocytes, compared to 40.1% live birth rate in women younger than 35 seeking ART [1]. Due to this low success with ART in older women and the increased delay in childbearing during the last 40 years, age-related decline in female fertility has become the largest and most difficult problem to be solved in the field of human reproduction.

Age-related decline in female fertility is mainly driven by ovarian aging, or diminished ovarian function associated with age [2]. Decline in ovarian function is clearly demonstrated by reduced response rates to ovarian stimulation during ART as women age, and by the restoration of high birth rate in older women using oocytes donated from young women [2]. The end point of ovarian aging, i.e., ovarian failure, is manifested as menopause. Menopause occurs at an average age of 51 and is associated with dramatic increase in many age-related diseases.

Diminished ovarian function is generally attributed to decreased quantity and quality of oocytes and their surrounding granulosa cells during ovarian aging, although the underlying mechanisms causing this age-related loss remain unclear. Transcription studies have suggested indirectly that some age-related changes in oocyte gene expression may involve epigenetic machinery [3, 4]. However, epigenetic change as a potential mechanism underlying ovarian aging process has never been directly investigated, even though epigenetic dysregulation has been proposed to play a critical role in aging and age-related diseases [5–7].

Loss of ovarian function is one of the most universal features of the aging phenotype in humans and other primates, thus the investigation of epigenetic mechanisms of ovarian aging addresses not only a fundamental question in human reproduction, but should also provide an efficacious system for the study of aging in general. Further, the easy accessibility of granulosa cells made possible by ART procedures, as well as the relative homogeneity to which these cells can be purified, make this an excellent system to study age-related epigenetic alterations. Granulosa cells remain dormant in ovaries for up to several decades while being subjected to frequent micro-environmental changes associated with ovulation; accordingly, epigenome alterations in these cells may truly reflect the interaction between the genome and environment.

Perturbations in DNA methylation have been described as a feature of aging in mammals [8–11] and are common in cancerous tissues [6–8, 12]. Such aging-associated perturbations can be shared among multiple cell types or have tissue-specific characteristics [8, 13–15]. Although initially described as an epigenetic mark for gene silencing [16, 17], DNA methylation has more recently been shown to have varying relationships with transcription dependent upon specific genomic contexts [18]. Until recently, the great majority of studies focused on methylation in the promoter region adjacent to the transcription start site (TSS), which blocks transcription initiation. Advances in genome-wide approaches have revealed that methylation in the gene body does not block transcription elongation [19, 20] and may have a role in regulating splicing [21, 22]. However, little is known about the relationship between methylation at the 3ʹ-end of the gene and transcription in animal or human cells.

We conducted the first mRNA-seq and genome-wide DNA methylation study of women with age-related differences in ovarian function, and found distinctive changes in DNA methylome and transcriptome structures in ovarian granulosa cells as women age and their ovarian functions deteriorate. DNA methylome features were linked to gene expression changes in many genomic regions, and an especially strong correlation was noted when the enrichment in methylation was mapped to the 3ʹ-end of the gene, i.e. in proximity to the transcription end site (TES).

## RESULTS

Gene transcription as well as genomic DNA methylation patterns in ovarian granulosa cells were compared between two groups of women: (i) *oocyte donors* (*n* = 20) who were young (age 26 ± 2.2 years) and had robust response to ovarian stimulation during assisted reproductive technology (ART) (mean number of oocytes retrieved = 25); versus (ii) *poor responders* (*n* = 20) who were older (age 40 ± 2.3 years) and responded poorly to ovarian stimulation during ART (oocytes retrieved ≤ 4 and peak estradiol level ≤ 1000 pg/ml). The first group served as healthy controls. The second group represented the majority of women in their early 40s who have the natural age-related decline of ovarian functions and therefore respond poorly to ovarian stimulation during ART. DNA methylomes were investigated using both Methylated DNA Capture followed by Next Generation Sequencing (MethylCap-seq) and Reduced Representation Bisulfite Sequencing (RRBS) methods.

### Transcriptome differences

Six individuals in each group were randomly selected and their poly-A (+) selected RNA libraries were indexed and sequenced on the same lanes (Gene Expression Omnibus Accession Number: GSE62093). Although these 12 individuals were randomly chosen, their transcriptomes fell into two distinct clusters that were consistent with their differences in age and ovarian function (Figure S1A). Both balanced and unbalanced permutation analyses, consisting of 1324 different possible sample combinations in the two groups, demonstrated that their transcriptome differences reflected biological differences between groups, rather than random individual differences (Figure S1B, S1C).

**Figure 1:**
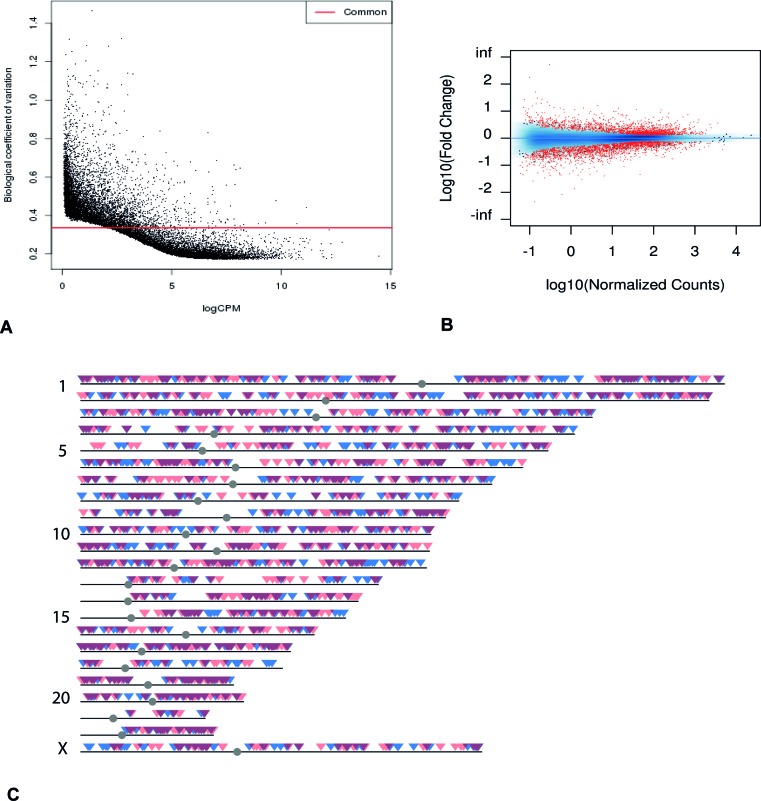
Transcriptome overview **(A)** Dispersion graph of all genes from mRNA-seq. Genes under the common coefficient variation threshold (red line) were included in further analysis by EdgeR (qCLM). **(B)** MA plot of all the genes from mRNA-seq. Genes in red showed statistically significant differential expression (FDR < 0.05). **(C)** Chromosomal distribution of genes with statistically significant differential expression. Blue: genes with increased expression in poor responder group. Red: genes with decreased expression in poor responder group. Dark red: overlapping regions.

Several bioinformatics tools were applied to analyze the transcriptome data, and these generated comparable results (see Methods for details). After eliminating genes with high variability among samples based on the dispersion graphs (Figure 1A), we identified 3397 genes that were differentially expressed (FDR < 0.05), with 1809 down-regulated in the poor responder group (Figure 1B, 1C). Among these differentially expressed genes, a number are known to be closely associated with ovarian function (e.g. *AMH* [23]*, TNFRSF11A, INHBB* [24]*, BMP4, BMP6, BMP7, GDF9, DICER1* [25]). For example, *TNFRSF11A* (also known as *RANK*) plays a key role in apoptosis [26] and is up-regulated in mice with environmental toxin induced ovarian failure [27]. GWAS studies have shown that SNPs of this gene are related to the age of menopause [28, 29]. The expression level of this gene was much higher in the poor responder group than the oocyte donor group (logFC = 1.79, FDR = 6.6 × 10^−3^).

IPA pathway analysis revealed these differentially expressed genes to be highly represented in multiple pathways (Figure S2), several of which are known key players in ovarian functions. For example, the polo-like kinase pathway, which was down-regulated in poor responders, plays a major role in cell cycle arrest of granulosa cells during their luteinization in the periovulatory period [30]. The G2/M cell cycle DNA damage checkpoint pathway, also down-regulated in the poor responder group, was shown to be significantly associated with the age of menopause in large-scale GWAS studies [31].

### Genome-wide DNA methylation analysis

A primary objective of our study was to investigate whether DNA methylation in granulosa cells may be an underlying mechanism contributing to (or in some instances protecting against) age-related changes in gene expression and ovarian function. To this end, we next compared DNA methylomes in ovarian granulosa cells from oocyte donors versus poor responders using two approaches: MethylCap-seq for broader genomic coverage, and RRBS for absolute quantification. (Gene Expression Omnibus Accession Number: GSE63470). Due to very limited amount of materials available from each poor responder, samples containing equal amounts of granulosa cell DNA were pooled from 10 individuals in each group. A second set of experiments pooling granulosa cell DNA samples from independent donor and poor responder groups (ten individuals each) was then performed. In addition, three technical RRBS repeats were performed with one paired set of DNA samples from donor and poor responder groups.

In analyzing the data from these experiments, it was important to establish interrelationships between the bisulfite- and affinity-enrichment-based approaches. Within 36 bp regions of direct overlap, CpG density-adjusted MethylCap-seq coverage and RRBS-measured methylation showed an approximately linear correlation (albeit with high scatter); (Fig S3 A, B) further, such localized 36 bp MeCAP data could be fitted to enrichment for encompassing 250 bp peak features (Figure S3 C, D). Although the first and second experiments were drawn from independent sets of individuals, 95.3% of those 250-bp regions that exhibited differential methylation between oocyte donors and poor responders (*n* = 16377) changed concordantly (Figure S4).

Due to intrinsic differences between the two methodologies, MethylCap-seq and RRBS methods generated strikingly different coverage with respect to age-related methylation patterns and change. CpG island overlap was found to be 3.5% and 44% for MeCAP peaks and RRBS regions, respectively. The median CpG methylation level for RRBS data was 9.5%, whereas that of MeCAP was 88% (as determined from the subset of RRBS data localized within MeCAP peaks).

**Figure 2:**
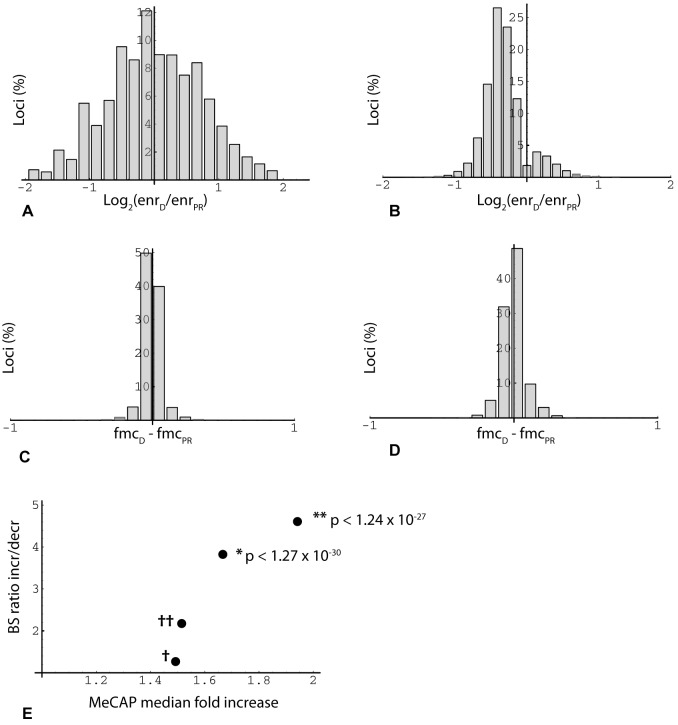
Overview of MethylCap-seq and RRBS datasets **(A)** Histogram of loci binned by average methylation fold change in two sets of MeCap-seq experiments. Minimum enrichment 5-fold for higher value in comparison (*n* = 2,534,732). X-axis: log_2_(average enrichment in oocyte donor group (enr_D_)/average enrichment in poor responder group (enr_PR_)); e.g., *x* = −1 means 2-fold increase in methylation in poor responder group compared to oocyte donor group. **(B)** Histogram of loci binned by methylation fold change. Minimum enrichment 15-fold for higher value in comparison and change significant (two-sided T test; *p* < 0.05) for duplicate MeCap-seq experiments (*n* = 15,783). **(C)** Histogram of RRBS results binned by change in fractional 5ʹ-methylcytosine (fmc). Data from first of triplicate RRBS datasets. In this and other figures, inclusion of loci (read positions) in the analysis required > = 2 CpGs (read length 36 bp) and read depth > = 20. X-axis: difference between oocyte donor group (fmc_D_) and poor responder group (fmc_PR_) (*n* = 221,331). **(D)** Histogram of RRBS results binned by average fmc change, where value was significant (two-sided t-test; *p* < 0.05) for triplicate RRBS datasets (*n* = 11,356). **(E)** Agreement in methylation change measurements between MeCap-seq and RRBS datasets. RRBS experiments yielded 33503 loci with consistent change (fmc_D_ - fmc_PR_) in triplicate technical repeats (increase/decrease (I/D) ratio 1.11 (17661/15842)). This reference ratio was compared (Fisher's exact test) to ratios obtained for RRBS locus subsets mapped within the boundaries of MeCap peaks. As an initial control (†), RRBS data from the first technical repeat were aligned within MeCap peaks with an I/D ratio of 1.02 (first experiment, minimum enrichment 2-fold; normalized from 1.12 by an iterative algorithm); the RRBS subset ratio returned by this filter was 1.27 (*n* = 58533). As a second control (††), RRBS data from triplicate repeats (reference set) were aligned within MeCap peaks that exhibited an increase in methylation in duplicate experiments (qualitative agreement); this filter yielded a RRBS subset ratio of 2.18 (*n* = 2302). To test for RRBS-MeCap agreement, RRBS loci that exhibited significant change in triplicate repeats (two-sided T test; increase/decrease ratio 1.23; *n* = 11356), were aligned within MeCap peaks that showed a significant increase (median 1.67) in duplicate experiments; this shifted the RRBS subset ratio upwards to 3.82 (*n* = 439; Fisher's exact test *p < 1.27 × 10^−30^). A similar test for agreement was done by selecting RRBS loci with significant change that aligned within MeCap peaks that displayed an increase of > = 1.5-fold (median 1.94) in duplicate experiments; here the RRBS subset ratio was shifted to 4.61 (*n* = 314; Fisher's exact test **p < 1.24 × 10^−27^).

**Figure 3:**
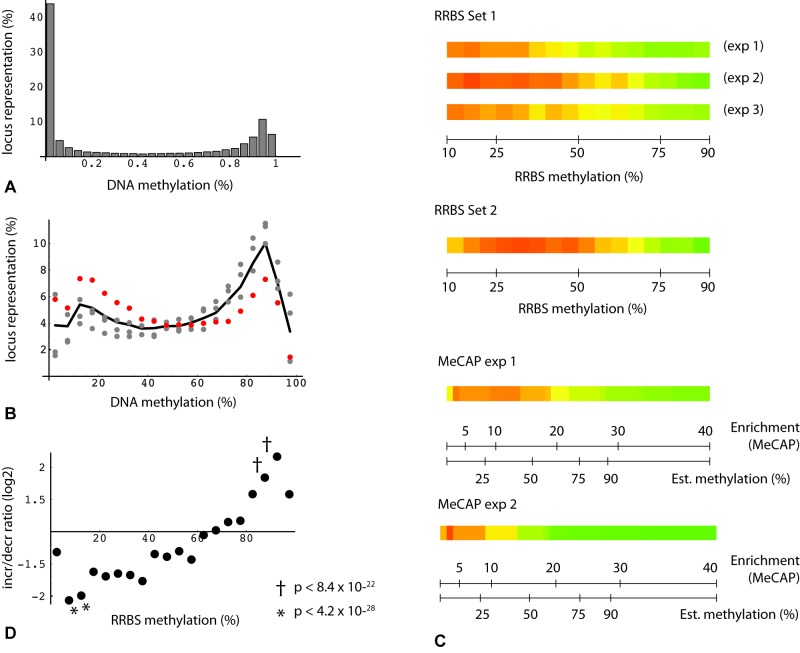
Bimodal distribution and change in DNA methylation levels **(A)** Percentage of loci binned by DNA methylation for all oocyte donor loci with consistent change in triplicate RRBS technical repeats (*n* = 33,503). **(B)** Percentage of loci binned by DNA methylation: plot restricted to subset of RRBS read positions with consistent change (fmc_D_ - fmc_PR_) > = 0.075 (*n* = 6,556). Gray points: 3 technical replicates from one set of donors. Red points: RRBS from second independent set of individuals. Black line: average from the four datasets. **(C)** Heatmap depiction of loci binned by oocyte donor enrichment. Green: increased methylation in poor responders relative to donors; red: decreased methylation in poor responders. Each binned value derived from minimum locus count of 200. **(D)** Plot of increase/decrease ratios as a function of binned fractional methylation values from oocyte donors (triplicate RRBS determinations from first set of individuals). Each binned value derived from minimum locus count of 20. Probability estimates (Fisher's test) represent increase/decrease (I/D) locus counts for binned subsets vs. reference set (I/D ratio 1.11 (17661/15842)).

An initially puzzling observation with respect to age-related change was that MethylCap-seq peaks, but not RRBS regions taken as a whole, tend toward hypermethylation. The MethylCap-seq-specific asymmetry is not evident where single experiments and all loci are considered (Figure 2A, 2C), but becomes prominent when requirements for higher enrichment and for consistent, statistically significant change in duplicate experiments are imposed (Figure 2B, 2D). The explanation for these observations relates to the very different genome coverage profiles of the two methods noted above. This became evident when we plotted read position representation vs. binned methylation levels from the oocyte donor group; in so doing, we noticed a bimodal distribution, with the highest values corresponding to either very low (10%) or very high (90%) levels of methylation (Figure 3A, 3B). We then asked whether this distribution might shift in the older poor responders, which led us to plot changes in methylation in older poor responder group compared to young oocyte donors as a function of donor methylation levels. As seen in Figure 3, within the range of 10% to 90%, all four RRBS and both MethylCap-seq datasets demonstrated a genome-wide age-related drift toward more extreme levels of methylation (Figure 3C). This trend could be most accurately quantified for triplicate-repeat RRBS data derived from the first set of individuals (Figure 3D); here, at a high level of methylation (85 to 95%, in green regions), most loci (67%) showed increased methylation in the poor responder group compared to young oocyte donors. By contrast, more loci (67%) showed decreased methylation in the poor responder group at a low level (5 to 15%) of methylation.

A key prediction from preceding analysis is that the change bias seen in the MethylCap-seq results will likewise be evident in RRBS results where the two datasets overlap, i.e., where RRBS read positions fall within MeCAP peaks. This is indeed the case (Figure 2E), with the asymmetry in RRBS data increasing in accord with greater stringency of the criteria for MeCAP peak change. We conclude that the MethylCap-seq results which reveal elevated methylation in poor responders are independently validated by the RRBS experimental data. Overall, these highly significant results illustrate a genome-wide age-related drift away from intermediate toward either high or low levels of methylation.

### Correlations between transcriptome and DNA methylome changes

Building on the foundation of consistent and distinctive patterns of change in both transcriptomes and DNA methylomes in granulosa cells from these two groups of women, we set out to determine how the age-related alterations in gene expression may be associated with epigenome structure at the level of DNA methylation. The most straightforward approach was to search for genes that exhibit both methylation and transcription change. This approach does yield examples, the most interesting identified in the present study being the *UHRF1* (Ubiquitin-like with PHD and ring finger domains 1) gene. *UHRF1* expression is down-regulated in the poor responder group (logFC = −1.44, FDR = 0.014). For a 450 bp peak spanning the eleventh and twelfth coding exons, increasing levels of methylation were found in concordant MethylCap-seq datasets, as well as in triplicate RRBS data within the peak (78% in the oocyte donor group, 92% in the poor responder group; *p* value < 0.028). At one CpG site (chr19:4950849 (Build37.2)) with significant change in these triplicate RRBS datasets (69~74% in the oocyte donor group, 87–94% in the poor responder group; *p* value < 0.0033), the methylation levels were also validated using EpiTYPER assay (71% in the oocyte donor group, versus 89% in the poor responder group). UHRF1 is recognized as a main “hub protein” involved in the fidelity and integration of epigenetic information, linking DNA methylation, histone modifications, and heterochromatin formation [32, 33]. Its over-expression plays an important role in the pathogenesis of ovarian cancer and various other cancers [34–36].

**Figure 4:**
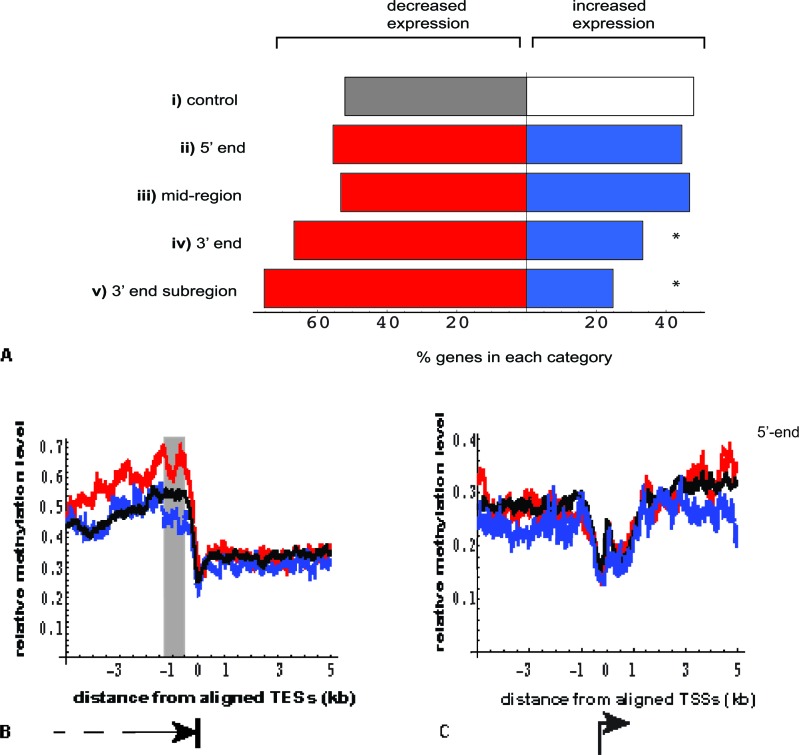
Relationship between location of DNA methylation enrichment in MeCap-seq data and the direction of gene expression change **(A)** Genes with significant differential expression (FDR < 0.05) were selected for overlap by 2kb regions with > = 5-fold MeCap enrichment; direction of expression change was then correlated with the gene subdomain to which methylated regions mapped ( *p* values calculated using Fisher's exact test, compared with the control.)
control: genes with significant expression change not overlapping methylation-enriched regions (*n* = 723 vs. 665, genes with decreased vs. increased expression in the poor responder group, respectively).5ʹ end: center of methylation enrichment located within gene, ≤ 2000 bp from TSS (*n* = 76 vs. 61; *p* > 0.05).mid region: center of methylation enrichment located > 3000 bp from either TSS or TES (*n* = 375 vs. 329; *p* > 0.05).3ʹ end: center of methylation enrichment located ≤ 2000 bp from TES (*n* = 178 vs. 89; *p = 1.3 × 10^−5^).3ʹ end subregion: center of methylation enrichment located within a window of −1300 to −500 bp from TES (*n* = 85 vs. 28; *p = 9.7 × 10^−7^). This subregion was chosen based on Figure 4b shaded area.
**(B)** Near the 3ʹ-end (TES), genes with decreased expression in poor responder group (red, *n* = 1464) showed increased MeCap enrichment, compared to genes with minimal expression changes (black, *n* = 7,315) and those with increased expression (blue, *n* = 1212). The window showing the greatest difference in enrichment was −1300 to −500 bp from the TES (gray rectangle). **(C)** Near the 5ʹ-end (TSS), no difference in MeCap enrichment was observed among genes with decreased expression (blue), increased expression (red), and minimal expression change (black).

Beyond examples such as *UHRF1*, we detected no overall statistical correlation between transcription and methylation change. Conversely, analyses of the two MeCap-seq datasets revealed, somewhat surprisingly, that DNA methylation enrichment alone had a strong statistical association with gene expression. Furthermore, as we examined the enrichment patterns within 2676 autosomal genes with highly significant differential expression (FDR < 0.05, |logFC| > 0.5), we found that the location of DNA methylation enrichment influenced its linkage to expression. Compared to control genes without enrichment in DNA methylation, genes with methylation enrichment at either the 5ʹ-end (≤ 2000bp from the Transcriptional Start Site (TSS)), or within the gene body (≥ 3000bp from the TSS and Transcriptional End Site (TES)), did not correlate with the direction of change in gene expression (Figure 4A). By contrast, an elevated percentage of genes with methylation enrichment at their 3ʹ-ends (≤ 2000bp from TES) showed decreased expression in the poor responder group (*p* <0.01) (Figure 4A).

To gain further insight into this correlation near 3ʹ-ends, we aligned MeCap-seq enrichment data to transcription end sites. This alignment revealed that 1464 autosomal genes with significantly decreased expression in the poor responder group had higher MethylCap-seq enrichment in proximity to TES, compared to genes that were up-regulated in this group or those with minimal change in expression (FDR > 0.05, |logFC| < 0.3) between the two groups (Figure 4B). The 3ʹ-end subregion with the highest enrichment differences and the strongest correlation with the down-regulation of gene expression localized within 1300 to 500bp upstream from the TES (Figure 4A subregion and 4B shaded area). The varied levels of methylation near 3ʹ-ends were in large part due to differential CpG contents among the three groups of genes (up-regulated, down-regulated, and minimal change in expression). Of note, such differences were not observed for aligned promoter regions, where the corresponding comparisons revealed methylation to be independent of the direction of gene expression change (Figure 4C).

**Figure 5:**
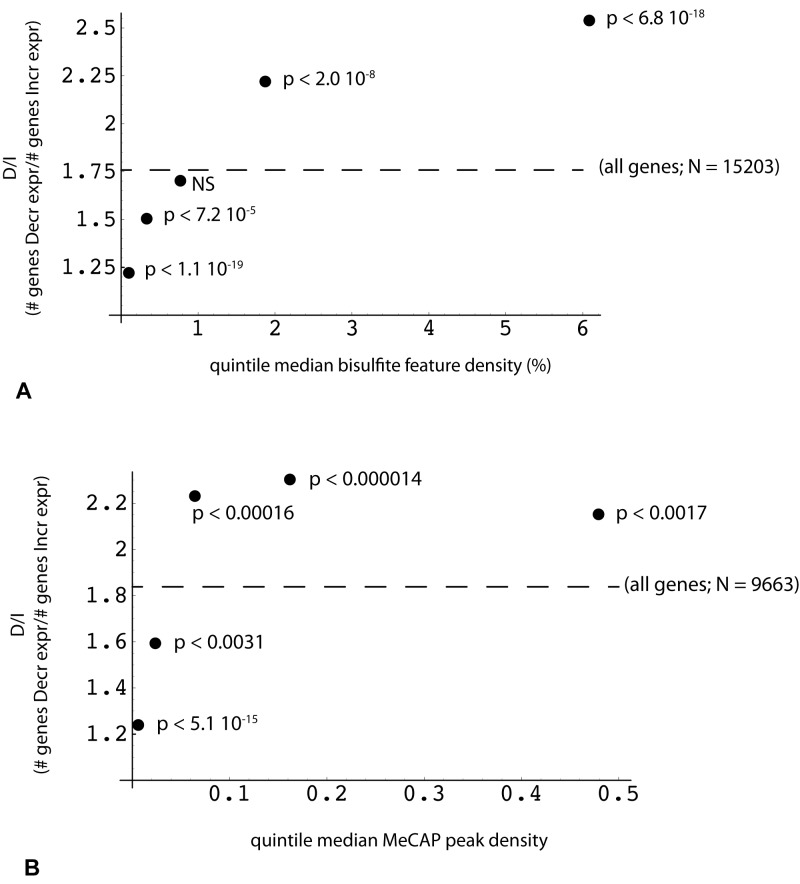
Reciprocal relationship between the change in gene expression and the density of DNA methylation peaks within the gene Methylome features (RRBS or MeCap) were mapped for overlap with one or more genes using a sparse array-based algorithm. Each gene with a valid expression value obtained by EdgeR analysis (*n* = 29878) was then associated with a list of overlapping methylome features (“all genes”, designated by dotted line; RRBS, *n* = 15203; MeCap, *n* = 9663). X axis: median value of **(A)** RRBS locus or **(B)** MeCap peak density (100 * locus or peak no. *width/gene length) within each quintile. Y axis: ratio (number of genes with decreased expression in poor responders: number of genes with increased expression) for corresponding quintile; *p*-values were calculated by comparison of quintile and relevant control (“all”) gene count pairs using Fisher's exact test.

Given MethylCap-seq results indicating a role for high CpG content in proximity to the TES, we sought to determine whether the RRBS data would be confirmatory. This could be demonstrated, but in the course of the analysis, two other important parameters linking transcriptome and genome/methylome structure came to light. The first of these relates to the density of bisulfite read positions ***(bsDens)*** embedded within gene transcription units, where bsDens is presumably a proxy for GC or CpG density. By plotting the ratio of down-regulated to up-regulated expression *(D/I ratio)* as a function of median bsDens values, it can be seen that D/I increases very significantly from the lowest to highest sorted bsDens quintiles (Figure 5A). By way of reference, the lowest quintile corresponds to median gene body coverage of GC-rich regions of 17.3% (where the minimal unit for GC-richness is > 50% over 100 bp). The highest bsDens quintile corresponds to 79.3% of gene bodies spanned by GC-rich domains. A somewhat comparable relationship is seen in the MethylCap-seq experiments, but only for lower MeCAP peak densities and with less significant *p* values (Figure 5B).

**Figure 6:**
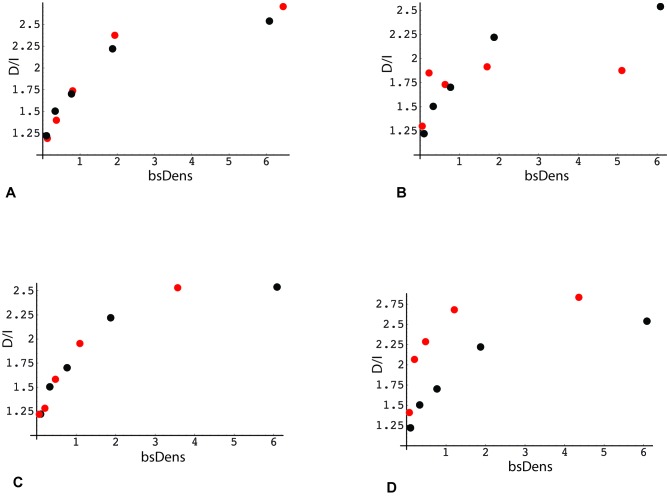
Association of the direction of gene expression change with bisulfite peak density and methylation levels in RRBS data (analysis 1) X-axis: Median RRBS locus density in each quintile. Bisulfite densities (bsDens) were calculated as: 100 * (number of bisulfite loci within the gene *36)/gene length in bp. Y-axis: Ratio (number of genes with decreased expression in poor responders: number of genes with increased expression) Black points (panels A – D): plot derived from RRBS reference gene set (same as Figure 5, panel A; *n* = 15203). **(A)** Red points: gene subset with average intragenic methylation level of RRBS loci < 0.5. **(B)** Red points: subset with average intragenic methylation level of RRBS loci > 0.5. **(C)** Red points: gene subset with RRBS loci localized within 5ʹ-end quartile of gene length, i.e., distance from TSS to the most 3ʹ-proximal locus less than 25% of total gene length. **(D)** Red points: subset with RRBS loci localized within 5ʹ-end quartile of gene length, i.e., distance from TSS to the most 5ʹ-proximal locus greater than 75% of total gene length.

The second parameter influencing D/I transcriptome ratios is gene body methylation. To obtain a preliminary evaluation of this parameter, we again plotted the D/I expression ratio as a function of median quintile bsDens values, but this time for subsets of genes with high vs. low methylation. The rationale for comparing quintile plots of gene subsets is that the contribution of the bsDens variable is effectively normalized, allowing bias from the latter determinant to be removed from the comparison. As seen in Figure 6A, B, these high and low methylation subsets diverge, albeit in a complex pattern. Similarly, we used the quintile plotting strategy to obtain a preliminary estimate of the role of bisulfite read density position, comparing the full gene set to a subset where all bsDens values are localized within the 3ʹ terminal quarter of gene length (Figure 6C, 6D). As shown from this plot, the D/I expression ratio is again elevated in association with TES proximity to GC-rich regions.

**Figure 7:**
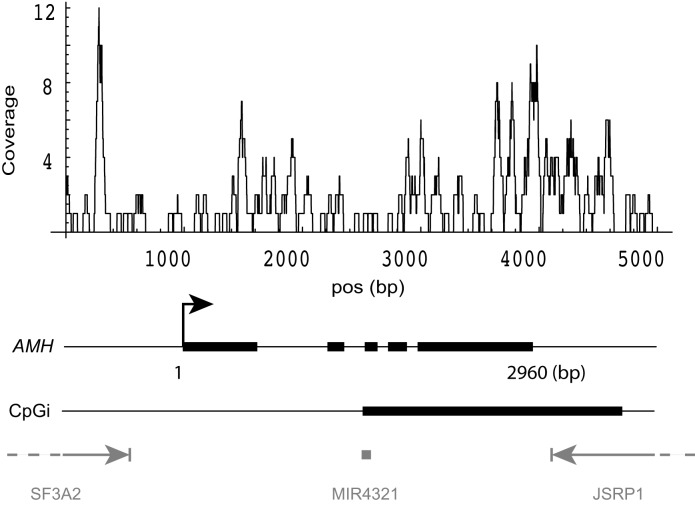
DNA methylation pattern of *AMH* gene DNA methylation coverage was calculated by summing the reads from both groups in two experiments. Gene structure of *AMH*: thick blocks are exons, thin lines within the gene are introns. CpGi: CpG island. Three neighboring genes are shown in gray.

It should be noted that a number of gene examples which have been studied in relation to age-related decline in ovarian function have properties consistent with the parameters identified above. In this context, the most interesting example is the Anti-Müllerian Hormone (*AMH)* gene, which has a very high GC content (96.6% of gene body is > 50% GC-rich), has a partially methylated CpG island close to its TES (Figure 7), and exhibits decreased expression in poor responders. AMH plays a key role in primordial follicle recruitment [23] and is a widely used marker for diminished ovarian reserve [37, 38]. Expression of *AMH* in the poor responder group was strikingly down-regulated (logFC = −2.92, FDR= 5.9 × 10^−13^) in our dataset. As determined by triplicate RRBS results, the CpG island that spans the gene 3ʹ-end was methylated at 13% vs. 8.7% in young donors and poor responders, respectively (*p* < 0.02).

An alternative strategy to evaluate potential linkage between transcriptome patterns and either methylation or TES proximity of GC-rich regions is to evaluate these variables while holding bsDens constant. This was accomplished by determining the gene body bsDens value for a queried gene subset, then randomly sampling high and low GC-rich pools from the reference gene set so as to yield a closely matching median bsDens value. The sampling procedure was then repeated 10,000-fold to generate non-parametric estimates for the significance of observed subset D/I expression values.

**Figure 8:**
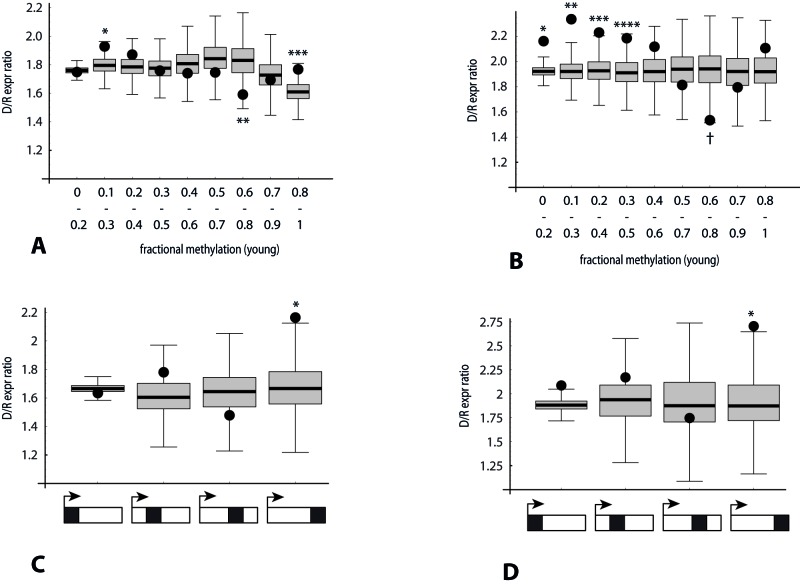
Association of the direction of gene expression change with bisulfite peak density and methylation levels in RRBS data (analysis 2) Y-axis: Ratio (number of genes with decreased expression in poor responders: number of genes with increased expression). Black dots: median observed ratio in each category. Box and whisker: distribution of ratios expected by chance in each category, obtained by repeated (10,000-fold) random sampling of two reference gene set pools (lower and higher bsDens) in order to approximate the observed bsDens for that category. See legend to Figure 5 for derivation of reference gene set (*n* = 15,203). *p*-values: fraction of sampling-derived ratios with greater deviation from the median than the observed ratio for that category. **(A)** Ten overlapping categories defined by average methylation level of RRBS loci within the gene (binned X-axis value ranges 0 to 0.2, 0.1 to 0.3, 0.2 to 0.4, etc.).*p < 0.02; **p < 0.019; ***p < 0.025. **(B)** Same as (A), except analysis restricted to genes with RRBS peak density > 0.5. *p < 0.0001; **p < 0.0001; ***p < 0.003; ****p < 0.012; ^†^p < 0.0016. **(C)** Four categories depicted below X-axis indicate RRBS read loci localized within (left to right): quartile of gene length proximal to 5ʹ-end, second quartile, third quartile, or quartile of gene length proximal to 3ʹ-end. *p*-value for 3ʹ-end quartile < 4.5 × 10−^3^. **(D)** Same as (C), except analysis restricted to genes with RRBS peak density > 0.5. *p*-value for 3ʹ-end quartile < 0.011.

Applying this strategy to investigate the role of RRBS-measured DNA methylation initially yielded only results of borderline significance (Figure 8A). However, restricting the analysis to a subset of genes with a moderately high RRBS read density (bsDens > 0.5, or in the upper 40^th^ percentile, which corresponds to 55% of GC-rich regions) yielded more striking methylation-related deviations (Figure 8B). From these plots we infer that for GC-rich regions distributed across the entire gene body, hypomethylated domains tend to correspond to increased D/I expression ratios, whereas a weaker converse effect is observed for moderately hypermethylated domains.

Lastly, the same repeated sampling strategy was applied to check for RRBS-based confirmation of the role of 3ʹ-end localization of GC-rich domains on age-related transcriptome differences. Here we determined D/I expression ratios and estimated probability values where all informative bisulfite read positions were confined to a specified gene body quartile. Only for the TES-proximal quartile subset were both an elevation of the D/I ratio and a significant positive correlation observed between down-regulation of gene expression and bisulfite read position (Figure 8C, 8D). This relationship was found to hold whether all genes were considered (D/I = 2.16; *p* < 0.0044), or the analysis was restricted the upper 40^th^ percentile subset for GC-richness (D/I = 2.7; *p* < 0.011).

## DISCUSSION

Unlike most other organs, human ovarian function declines sharply and uniformly from the third decade, and all major functions are completely lost by the early fifth decade. The postmenopausal phase makes up a large proportion of a woman's life span and has direct impact on age-related diseases. Beyond this impact, the uniform age-related decline in ovarian function and early-onset end point in ovarian aging make human ovarian granulosa cells an excellent experimental system for aging research. The high degree of agreement among independent sets of individuals in our study supports this conclusion.

Comparisons of both transcriptome and DNA methylation patterns in granulosa cells from older women with natural age-related decline in ovarian function (poor responders) to those from young healthy donor controls revealed clear differences between the two groups. We found that the direction of methylation change can be understood, at least in part, as a function of young donor (control) methylation levels; thus, increased methylation is predominant at more highly methylated donor sites, whereas decreased methylation occurs frequently at poorly methylated donor sites. This genome-wide age-related drift toward more “extreme” levels of methylation has not, to our knowledge, been reported previously.

Using the set of bioinformatics tools developed for this study, we were able to link DNA methylation levels to gene expression changes across the transcriptome, and thereby to identify several unique characteristics of these associations on the genomic level. One major finding was that the strongest correlation between DNA methylation and age-related variation in gene expression was observed when the 3ʹ-end of the gene was GC-rich, as demonstrated by high methylation enrichment in the MeCap-seq data or peak density in the RRBS datasets. This association was supported by three lines of evidence: (i) among all genes with significant differential expression (FDR < 0.05, |logFC| > 0.5), genes with methylation enrichment at the 3ʹ-end were more likely to be down-regulated in the poor responder group (Figure 4A); (ii) genes that were down-regulated in the poor responder group showed higher levels of MeCap-seq enrichment in proximity to TES, compared to those with up-regulation or no change (Figure 4B); (iii) when the RRBS peaks were distributed solely in the 3ʹ-end quartile of gene length, a significantly higher percentage of genes down-regulated in the poor responder group, as compared to the rest of the genes (Figure 6C, D; Figure 8C, 8D). The relationships between methylation level and gene expression change were more complex (Figure 6A, B; Figure 8A). When only genes with moderate to high GC-richness were considered (i.e. bsDens > 40^th^ percentile), low methylation levels (< 50% methylated) were clearly associated with an elevated percentage of down-regulated genes (i.e., D/I ratio > 2) (Figure 8B).

Although methylation of the promoter region is well established as a silencing mechanism, and the gene body has recently been recognized as a major mechanism for regulating gene expression in many tissues [18, 39], methylation at 3ʹ-end of the gene has received little attention except in plants [40]. One earlier study did show that increased methylation at 3ʹ-UTR of a tumor suppressor gene *p15^INK4b^* resulted in decreased transcription of this gene in primary lymphoma [41]. Another study in colorectal cancer demonstrated tumor specific DNA methylation of CpG islands located in 3′ exons was associated with up-regulation of *IPF1/PDX1* and *OTX1* gene expression [42]. In a recent manuscript, Yu *et al*. reported a group of 3ʹ CpG islands that gained methylation during human embryonic stem cell differentiation, and regulated transcriptional activation via a CTCF-blocking mechanism [43]. With respect to age-related gene expression change, the relationship to 3ʹ-end methylation has not, to our knowledge, been previously explored on the genomic level in animal or human cells.

It is known that many regulatory factors associated with mRNA 3ʹ-end formation also collaborate in the initiation of transcription, and several of these factors are involved in setting up appropriate chromatin structure to facilitate efficient transcriptional elongation and termination [44, 45]. An aging-related example that may illustrate such mechanisms is the regulatory element AE3ʹ, found in the 3ʹ-UTR of *FIX*, which is responsible for an age-related increase in coagulation factor IX mRNA levels [46, 47]. Our data, however, are equally consistent with the possibility that gene 3ʹ-ends may play a role in complex RNA processing or RNA export regulatory networks, and may thereby contribute to altered gene expression associated with aging. It is also possible that GC-richness in proximity to TES, or elsewhere within the gene body, may result in transcription blockage, since it has been demonstrated *in vitro* that GC-rich homopurine-homopyrimidine stretches produce such blockage through formation of R-loops [48]. R-loops have been associated with genome instability [49], and it is plausible that they could similarly promote epigenome instability.

*AMH*, a key gene in age-related decline in ovarian function, stood out as a striking example linking the age-related down-regulation of gene expression and the partial, low-level methylation in such GC-rich regions spanning 3ʹ-ends. This link between DNA methylation in proximity to TES and gene expression may be mediated by as yet unidentified sequence motifs, or may indicate that methylation levels can serve as informative marks for the involvement of other regulatory mechanisms such as nuclear architecture, chromatin structures containing Polycomb group family members, transcription factors such as CTCF [43], or non-coding RNA. Further investigations are needed to determine how genome and epigenome structure near the 3ʹ-end of the gene interact with these or other elements in the regulatory network of gene expression.

In conclusion, using next-generation sequencing approaches, we linked, for the first time, age-related decline of ovarian functions to distinctive gene expression changes and DNA methylation status in human ovarian granulosa cells. A genome-wide age-related drift toward more “extreme” levels of methylation was observed in both MeCap-seq and RRBS datasets. More importantly, we discovered a strong correlation between gene expression change and DNA methylation enrichment in proximity to TES, which has not previously been described on a genomic level in animal or human cells. Higher intragenic levels of GC-richness, as indicated by methylation enrichment in MeCap-seq or BS peak density in RRBS, especially when concentrated at gene 3ʹ-ends, were associated with reciprocal changes in gene expression.

## METHODS

### Ethics

All human materials used in this study were received under approval of the Office of Human Subjects Research at *Eunice Kennedy Shriver* National Institute of Child Health and Human Development, National Institutes of Health.

### Sample collection and cell purification

Follicular fluid samples were collected from Shady Grove Fertility Research Center. At the time of oocyte retrieval, after the oocytes were collected for continued ART treatment per standard ART laboratory protocol, the remaining follicular fluid was collected rather than being discarded. Follicular fluid samples were collected anonymously from two groups of women: oocyte donors, and poor responders (oocytes retrieved ≤ 4 and peak estradiol level ≤ 1000 pg/ml).

Ovarian granulosa cells were purified from each follicular fluid sample with similar methods as previously described [50]. Briefly, samples were placed on a Ficoll-paque gradient and centrifuged for 20 min at 900 × g at room temperature. The interphase was collected, and washed in DPBS. Collagenase was added for 4 minutes to disperse the cell clumps. After the cells were washed again with DPBS, CD45 labeled Dynabeads (Invitrogen, CA) were used to deplete leukocyte contaminations. The purity of each granulosa cell preparation was confirmed with flow cytometry (FACSCalibur; BD Biosciences, CA) using anti-CD45 (Miltenyi Biotec, CA) and/or anti-FSHR antibody (Assay Biotechnology, CA) (Figure S5). Over 95% purity was achieved for all granulosa cell samples before being used in subsequent experiments.

### RNA extraction and sequencing

Total RNA was extracted from purified granulosa cells using RNeasy Micro kit (Qiagen). RNA integrity and concentration were measured using RNA 6000 Nano kit (Agilent) on Agilent 2100 Bioanalyzer. All samples used for RNA-seq had RNA integrity number (RIN) of 9 or above.

For each sample, an mRNA sequencing library was prepared from 150ng of total RNA following Illumina TruSeq RNA low-throughput protocol. Briefly, poly-A containing mRNA was purified and fragmented. After cDNA synthesis, ends were repaired and adenylated. Indexed RNA adapters were ligated. All 12 indexed RNA-seq libraries were pooled and loaded on the same three lanes on Illumina HiSeq2000 for sequencing. The mRNA library and sequencing was done at NIH Intramural Sequencing Center (Rockville, MD). Approximately 40 million paired-end reads were achieved for each sample.

### Methylated DNA capture followed by next-gen sequencing (MethylCap-seq)

Genomic DNA was extracted from purified granulosa cells using QIAamp DNA mini kits (Qiagen). In each independent set of experiments, equal amounts of genomic DNA were pooled from 10 different individuals in each group (oocyte donors versus poor responders), after sonication and before MethylCap assays. We used 1–3 mg of fragmented DNA after pooling for each MethylCap assay. Methylated DNA Capture was performed as previously described [51, 52] by using MethylCap kit (Diagenode, NJ). Briefly, methylated DNA fragments were captured by incubating 2 μg of MethylCap protein (His6–GST–MBD, Diagenode) with 1 μg of fragmented DNA in binding buffer at 4^o^C for 2 hours. Washed magnetic beads were then incubated with the capture reaction in binding buffer at 4^o^C for 1 hour. After the bead–GST–MBD–DNA complexes were washed, the captured DNA fragments were eluted with High Elution Buffer. Input DNA was also treated with High Elution Buffer. The enrichment of captured DNA after MethylCap kit was confirmed by comparison to the input DNA with qPCR using human H19 and UBE primer pairs (Eurofins MWG Operon).

Sequencing libraries were prepared following Illumina ChIP Sequencing protocol. The DNA was end-repaired and ligated with Illumina sequencing single ended adaptors as described previously [51, 52]. After ligation, the DNA was enriched by 18 cycles of PCR with primers complementary to the single ended adaptor sequences followed by agarose gel size selection and purification.

For each MethylCap sequencing library, one to two lanes of 36 bp single-end sequencing were performed on the Illumina Genome Analyzer II according to the manufacturer's protocol at NIH Intramural Sequencing Center (Rockville, MD). Each sample achieved approximately 40 million reads.

### Reduced representation bisulfite sequencing (RRBS)

We based our RRBS method on two previously published protocols [53, 54]. Briefly, 1 ug of genomic DNA from each pool of purified granulosa cells (see MethylCap-seq method section for details) was digested with MspI restriction enzyme (New England Biolabs [NEB]). The DNA samples were end-repaired, ligated to Illumina methylated DNA adaptors and separated on agarose gel to isolate 150 ~ 340 bp fragments. DNA was then bisulfite converted using EZ DNA Methylation Kit (Zymo Research) and amplified by PCR.

Each RRBS library was sequenced on two lanes on the Illumina Genome Analyzer II sequencer according to the manufacturer's protocol at the NIH Intramural Sequencing Center (Rockville, MD). Each sample achieved approximately 40 million reads.

### EpiTYPER assay

Quantitative high-throughput DNA methylation analysis was done by MassARRAY system as described elsewhere [55], using Sequenom MassARRAY quantitative methylation analysis system. Briefly, genomic DNA was isolated and treated with bisulfite as described above. Bisulfite treated DNA was amplified using nested primers covering CpG sites within the *UHRF1* gene, spanning from Chr19:4950704 to Chr19:4950993. The primer sequences are available upon request. After Shrimp Alkaline Phosphatase treatment, the PCR products were used as a template for *in vitro* transcription and RNase A Cleavage for the T-reverse reaction as per manufacturer's instructions (Sequenom hMC). The samples were desalted and spotted on a SpectroCHIP (Sequenom), followed by spectral acquisition on a MassARRAY Analyzer (Sequenom) at Einstein Epigenomics Core Facility. The resultant methylation calls were performed by the EpiTyper software v1.0 (Sequenom) to generate quantitative results for each CpG site.

### Bioinformatics analysis

Transcriptome data was analyzed using STAR alignment followed by EdgeR [56, 57] or DESeq, or analyzed with the combination of Tophat, Cufflinks [58], and EdgeR. These different methods yielded comparable results. The gene expression results shown in this paper were generated from STAR/EdgeR (quantile-adjusted conditional maximum likelihood (qCML) method) pipeline. Ingenuity pathway analysis (IPA, http://www.ingenuity.com/) was used to identify biological networks that were over-represented in the list of genes with significant expression changes.

Reads from MeCap-seq experiments were aligned to human genome hg19 using Bowtie [59]. Bismark [60] was used for read mapping and methylation calling in RRBS datasets. Analyses of RRBS data were limited to reads that covered at least 2 CpG sites. Additional analyses were done using Perl scripts and C programs, which are available upon request (to B.H.). The gene annotation algorithms developed by B.H. for overlapping features at each genomic location are shown in the schematics (Figure S6). This set of programs allowed for detailed annotations at each genomic position, which were then used for fast searches and simultaneous comparisons of multiple genomic features.

This study utilized the high-performance computational capabilities of the Helix Systems (http://helix.nih.gov) and the Biowulf Linux cluster (http://biowulf.nih.gov) at the National Institutes of Health, Bethesda, MD.

## SUPPLEMENTARY FIGURES


