# Supplementary material for: DNA methylome and transcriptome sequencing in human ovarian granulosa cells links age-related changes in gene expression to gene body methylation and 3ʹ-end GC density
Source: Oncotarget. 2015 Feb 17;6(6):3627–43. doi: 10.18632/oncotarget.2875 (PMC4414142; doi:10.18632/oncotarget.2875)
Supplement: Supplementary file 1 [file oncotarget-06-3627-s001.pdf]

SUPPLEMENTARY FIGURES

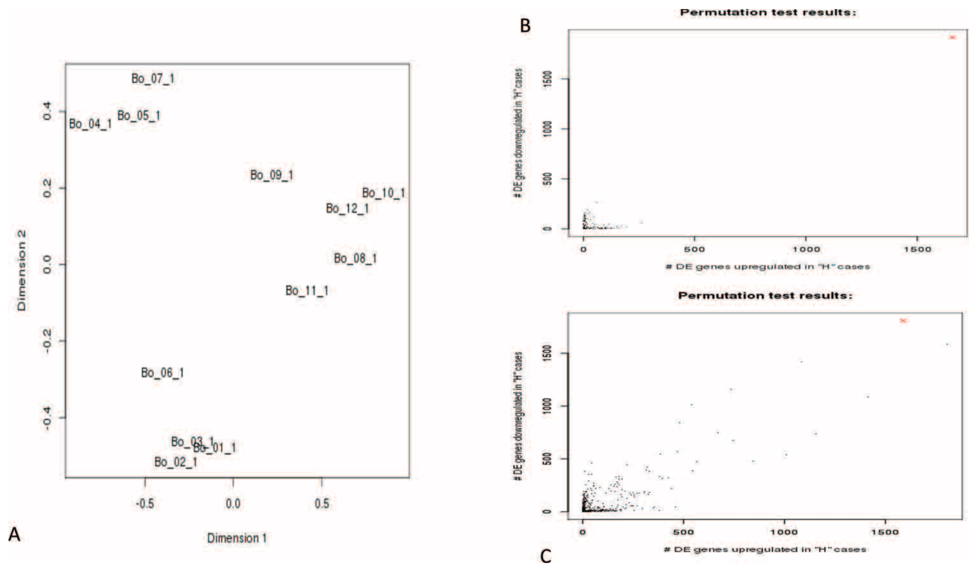

Supplementary Figure S1: Differences in mRNA-seq profiles are due to biological differences between the two groups. (A) Multi-dimensional scaling plot: The 12 individuals clustered into two groups that are consistent with their differences in age-related ovarian functions, except for one sample. **(B)** Balanced and **(C)** unbalanced permutation test showed that the highest number of differentially expressed genes was generated from the combination of experimental groups, rather than from other random combinations.

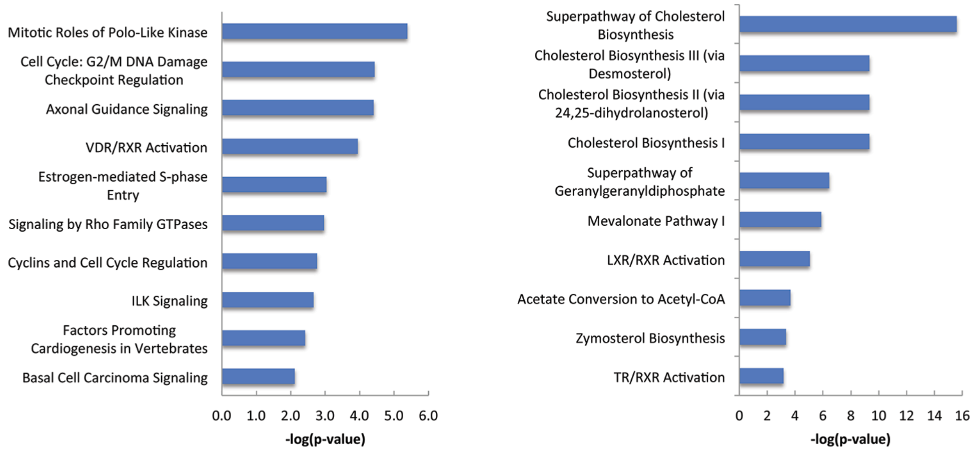

Supplementary Figure S2: Top 10 pathways that differentially expressed genes utilized (IPA Software, //www.ingenuity.com). Pathways involving genes with decreased (left) and increased (right) expression in poor responders.

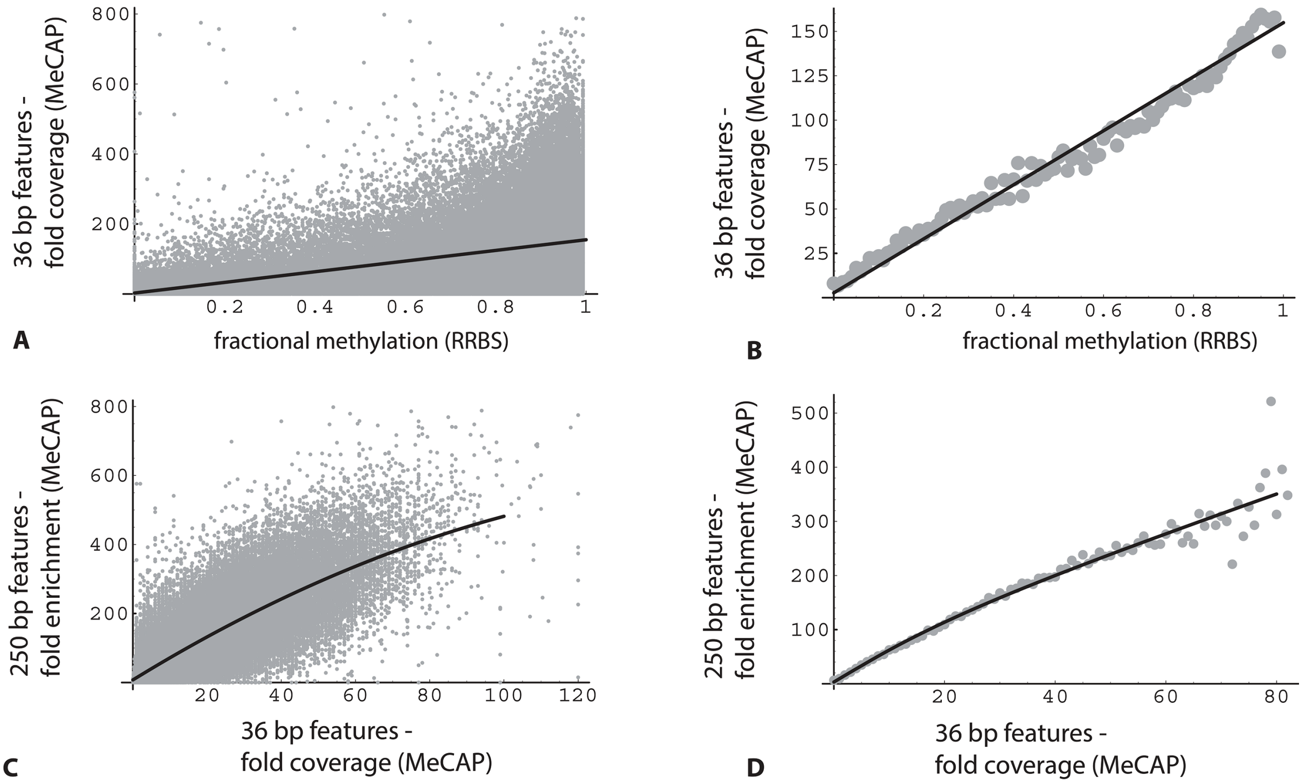

Supplementary Figure S3: Interrelationships between the bisulfite- and affinity-enrichment- based approaches (A) Each dot is a 36 bp region with RRBS data localized within MeCAP peaks. X-axis: average level of methylation in RRBS datasets. Y-axis: average fold coverage of each region in MethylCap-seq datasets. **(B)** Each dot is a 36 bp region with coverage in all MethylCap-seq and RRBS datasets. X-axis and Y-axis are the same as in A). **(C)** Each dot is a 36 bp region with RRBS data localized within MeCAP peaks. X-axis: average fold coverage of each region in MethylCap-seq datasets. Y-axis: average fold coverage (or enrichment) in the 250 bp sliding window that encompasses each 36 bp region. **(D)** Each dot is a 36 bp region with coverage in all MethylCap-seq and RRBS datasets. X-axis and Y-axis are the same as in A).

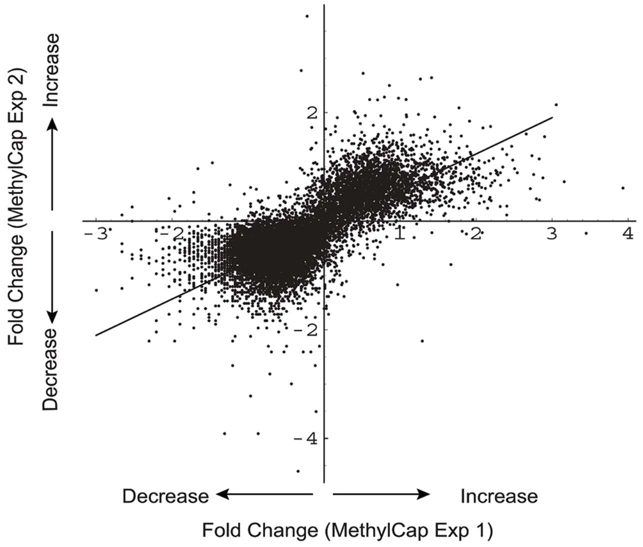

Supplementary Figure S4: Experimental agreement of MeCap-seq data. The first and second experiments were drawn from independent sets of individuals. Each dot represents a 250-bp region that exhibited differential methylation between oocyte donors and poor responders (n = 16377).

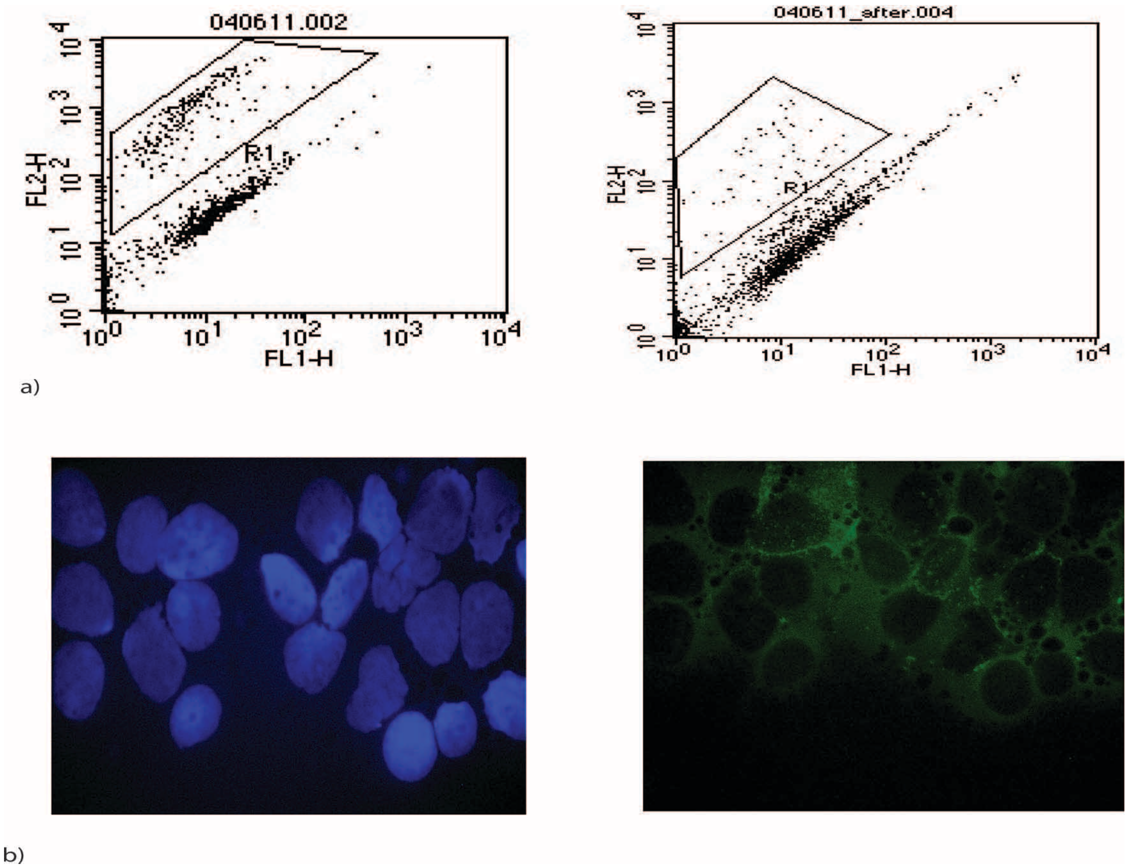

Supplementary Figure S5: Purification of granulosa cells. (a) FACS analysis of a granulosa cell sample before (left) and after (right) depletion using anti-CD45 labeled magnetic beads. Gated regions show that the percentage of leucocyte impurity decreases from 14% to 0.4%. **(b)** Immunofluorescent staining of a purified granulosa cell sample with DAPI (left) and granulosa cell specific FITC-labeled anti-FSHR antibody (right).

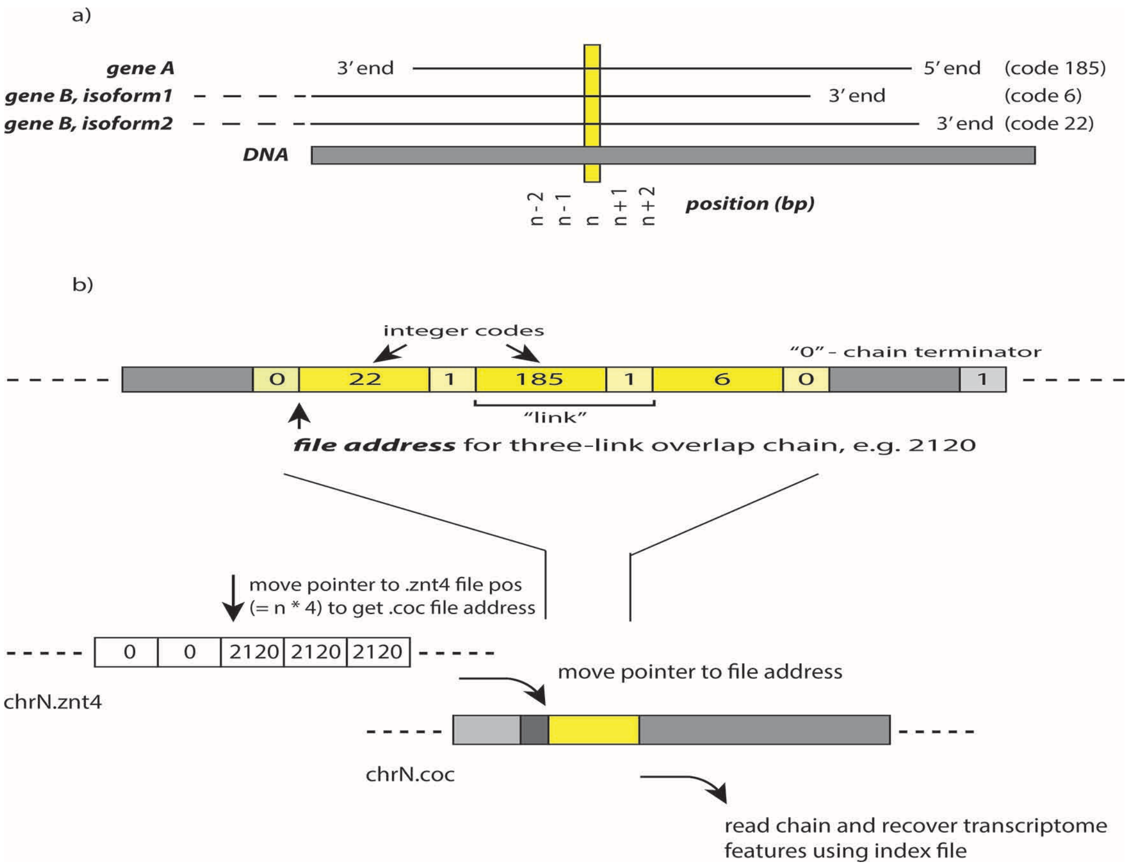

Supplementary Figure S6: Schematic for computer algorithms used to overlap multiple features at each genomic location. **(a)** An example of a genomic location (n) and its multiple associated features, such as gene A, gene B isoform 1, gene B isoform 2. Each feature is assigned with an integer code, such as 185, 6, 22, respectively, in the concatenated annotation file. **(b)** The set of algorithms for locating n and recovering its associated features. Three files are needed: annotation file (znt4), concatenated file (coc), and index file.

- Step 1. Move file pointer to chrN.znt4 file position (= n*4), to get address in chrN.coc file, eg. 2120.
- Step 2. In chrN.coc file, move pointer to file address 2120, which is associated with a 3-link chain. Each link represents a feature. Two links are separated by an integer "1". Two chains are separated by an interger "0".
- Step 3. Using index file, the integer codes recovered from Step 2 can be related to respective features.
